# Supplementary figures and images for: Functional characterization of the GhNRT2.1e gene reveals its significant role in improving nitrogen use efficiency in Gossypium hirsutum
Source: PeerJ. 2023 Mar 28;11:e15152. doi: 10.7717/peerj.15152 (PMC10064996; doi:10.7717/peerj.15152)

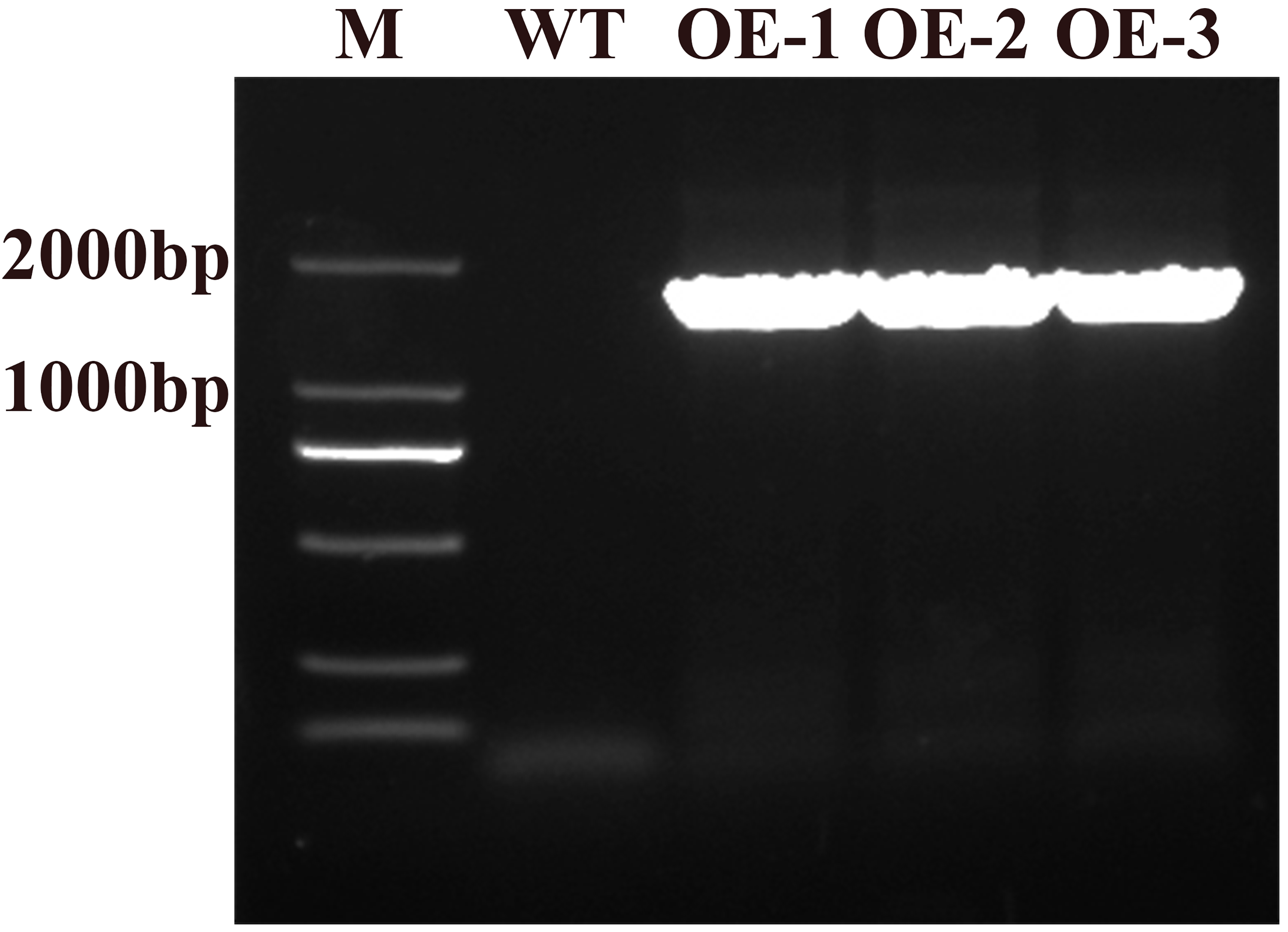

Supplement: Supplemental Information 7 — Genomic DNA from three T2 generation overexpressed lines was examined by PCR. “WT” represents the wild type; “OE” represents overexpression line. [file peerj-11-15152-s007.png]
